# Lung ultrasound: a new tool for the cardiologist

**DOI:** 10.1186/1476-7120-9-6

**Published:** 2011-02-27

**Authors:** Luna Gargani

**Affiliations:** 1 Institute of Clinical Physiology, National Research Council of Pisa, Italy

## Abstract

For many years the lung has been considered off-limits for ultrasound. However, it has been recently shown that lung ultrasound (LUS) may represent a useful tool for the evaluation of many pulmonary conditions in cardiovascular disease. The main application of LUS for the cardiologist is the assessment of B-lines. B-lines are reverberation artifacts, originating from water-thickened pulmonary interlobular septa. Multiple B-lines are present in pulmonary congestion, and may help in the detection, semiquantification and monitoring of extravascular lung water, in the differential diagnosis of dyspnea, and in the prognostic stratification of chronic heart failure and acute coronary syndromes.

## Background

### Sonographic prejudices: the history of lung ultrasound

**Figure 1:**
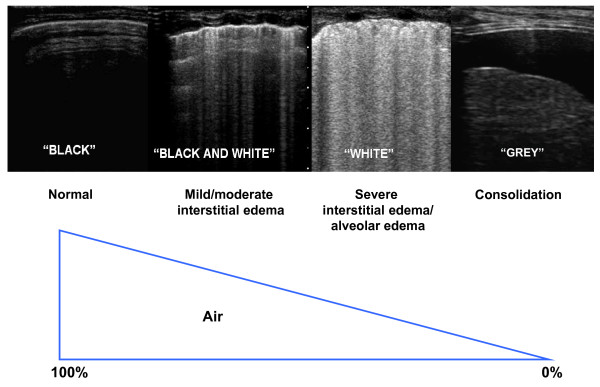
**Physical basis of lung ultrasound**. The less air is in the lung, the easier is the detection of lung abnormalities by ultrasound.

For many years ultrasound has not been employed for the evaluation of the lung [1]. All diagnostic ultrasound methods are based on the principle that ultrasound is reflected by an interface between media with different acoustic impedance. In normal conditions, with aerated lungs, the ultrasound beam finds the lung air and no image is visible, because no acoustic mismatch may reflect the beam, which is rapidly dissipated by air [2]. The only detectable structure is the pleura, visualized as a hyperechoic horizontal line, moving synchronously with respiration (see additional file 1). When the air content decreases - as in pulmonary edema, pulmonary fibrosis, etc. - the acoustic mismatch needed to reflect the ultrasound beam is created, and some images appear. In the presence of extravascular lung water (EVLW), the ultrasound beam finds subpleural interlobular septa thickened by edema. The reflection of the beam creates some comet-tail reverberation artifacts, called B-lines or ultrasound lung comets. A B-line is a discrete, laser-like, vertical, hyperechoic image, that arises from the pleural line, extends to the bottom of the screen without fading, and moves synchronously with respiration. Multiple B-lines are the sonographic sign of lung interstitial syndrome, and their number increases along with decreasing air content (see additional file 2). When the air content is further decreased, such as in lung consolidations, the acoustic window on the lung becomes completely open, and the lung may be directly visualized as a solid parenchyma, as the liver or the spleen (figure 1). Consolidations may be then measured and followed-up.

There are some anectodical reports on B-lines since the eighties [3,4]. In 1994, Targetta firstly described the presence of B-lines in diseased lungs [5]. But it was Daniel Lichtenstein, a French intensivist, who established for the first time the 2 main structural correlates of B-lines, comparing ultrasound findings with chest computed tomography (CT) [6]. CT data showed that B-lines were correlated to the thickening of subpleural interlobular septa in pulmonary interstitial edema, and to the fibrotic thickening in pulmonary fibrosis. The modern era of lung ultrasound (LUS) was born. It is true indeed, that LUS had already been employed since many years for the evaluation of pleural effusion (PE), but the acknowledgement of the information provided by artifacts represented a completely new approach. In 2004, Picano and Jambrik, in our laboratory, brought LUS from the Intensive Care Unit to the Cardiology Department, describing the correlation between EVLW assessed by chest X-ray, and the number of B-lines detected by LUS [7]. In the following years, experimental [8,9], clinical [10-14], and methodological [15] validation of B-lines have been provided.

## Methodology

**Figure 2:**
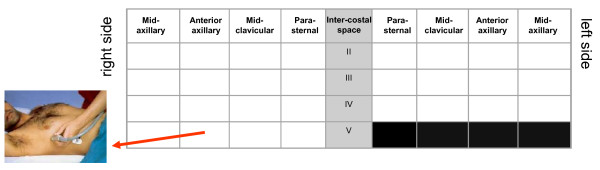
**Methodology for lung ultrasound evaluation**. Thoracic scanning areas for semiquantitative assessment of B-lines. (Modified from Jambrik et al, 2004 [7]).

**Figure 3:**
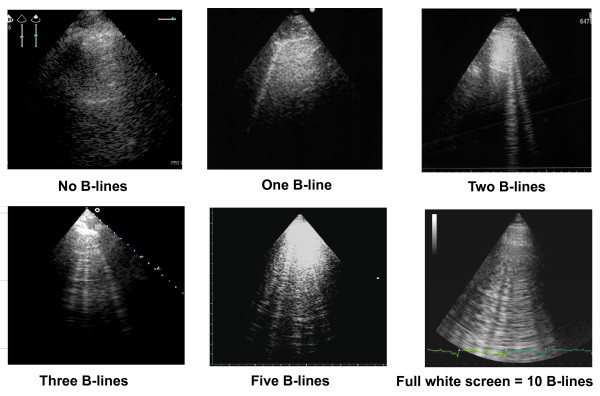
**How to enumerate B-lines**. Each hyperechogenic vertical stripe, spreading from the pleural line and extending to the edge of the screen, is a B-line. When using a cardiac probe, a whole white screen is considered as corresponding to a plateau value of 10 B-lines.

LUS examination can be performed using any commercially available 2-D scanner, with any transducer (phased-array, linear-array, convex, microconvex). There is no need for a second harmonic or Doppler imaging mode. The examination can be performed with any type of echographic platform, from fully equipped machines to pocket size ones [15]. Patients can be in the near-supine, supine or sitting position, as clinically indicated [16]. All the chest can be easily scanned by ultrasound, just laying the probe along the intercostal spaces. However, some specific methods have been proposed: ultrasound scanning of the anterior and lateral chest may be obtained on the right and left hemithorax, from the second to the fourth (on the right side to the fifth) intercostal spaces, and from the parasternal to the axillary line, as previously described [7,17]; (figure 2). Other approaches have been proposed, for instance by Volpicelli et al. [10], with evaluation of 8 scanning sites, 4 on the right and 4 on the left hemithorax. When assessing B-lines - the most informative LUS sign for the cardiologist - the sum of B-lines found on each scanning site yields a score, denoting the extent of extravascular fluid in the lung. In each scanning site, B-lines may be counted from zero to ten. Zero is defined as a complete absence of B-lines in the investigated area; the full white screen in a single scanning site is considered, when using a cardiac probe, as corresponding to 10 B-lines (figure 3). Sometimes B-lines can be easily enumerated, especially if they are a few; whereas, when they are more numerous, it is less easy to clearly enumerate them, since they tend to be confluent. In this situation, in order to obtain a semiquantification of the sign, one can consider the percentage of the scanning site occupied by B-lines (i.e. the percentage of white screen compared to black screen) and then divide it by ten (figure 3). For clinical purposes, B-lines may be categorized from mild to severe degree, similar to what is done for most echocardiographic parameters [16], (table 1). B-lines have a very satisfactory intraobserver and interobserver variability, around 5% and 7%, respectively [7].

**Table 1:** Scoring of B-lines

Score	Number of B-lines	Extravascular lung water
0	≤ 5	No sign
1	6 - 15	Mild degree
2	16 - 30	Moderate degree
3	> 30	Severe degree

(Modified from Picano et al, 2006 [16]).

## Clinical applications

### Heart failure

#### Diagnosis

In a 1994 review on the assessment of EVLW, Lange stated that «The possibility to detect pulmonary edema before it becomes clinically apparent, is so inherently attractive that the effort to develop and validate such technique still continues after many years of tireless and relatively unrewarding attempts» [18]. Chest X-ray remains by far the best and most used screening test for the detection of pulmonary edema, but it is often difficult to interpret and imprecise, and with high interobserver variability [19]. The absence of chest X-ray findings does not exclude the presence of a high pulmonary capillary wedge pressure (PCWP) [20]. According to recent 2009 AHA/ACC guidelines, serial chest X-rays are not recommended in the assessment of pulmonary congestion in chronic heart failure (HF), since they are too insensitive to detect but the most extreme changes in the fluid status [21]. Direct measurement of PCWP via catheterization is the gold standard to evaluate hemodynamic congestion, but its invasive nature limits clinical utility. Thus, because the current technology for measuring pulmonary edema can be inaccurate (chest X-ray), cumbersome (nuclear medicine and radiology techniques), or invasive (indicator dilution), there is great potential for a technology that could quantify pulmonary edema non-invasively in real time, with a radiation-free and portable method.

B-lines have been proposed as a reliable ultrasound technique for the assessment of pulmonary congestion in HF patients. The number of B-lines increases with worsening New York Heart Association (NYHA) functional class [14]. Sonographic B-lines are related to radiographic Kerley B-lines and lung water score on chest X-ray [7], to EVLW measured invasively by the thermodilution method [9], and to the severity of diastolic dysfunction, for any given level of systolic dysfunction [14]. B-lines are useful for the differential diagnosis of cardiogenic versus non-cardiogenic dyspnea. Lichtenstein et al., firstly described that B-lines could differentiate acute cardiogenic pulmonary edema from exacerbation of chronic obstructive pulmonary disease (COPD), since B-lines were present in all patients with cardiogenic edema, whereas 24 of the 26 patients with exacerbation of COPD had no B-lines, with a sensitivity of 100% and a specificity of 92% [12]. These data were further confirmed by our group, as we found that B-lines are reliable in predicting the cardiogenic origin of dyspnea, with an accuracy comparable to natriuretic peptides [11]. B-lines could be a plausible alternative in acute settings where natriuretic peptide analysis is not available, or when there is no time enough to perform it, as in patients with rapidly developing acute respiratory failure. Moreover, they could aid when natriuretic peptides levels are in the "grey zone".

**Figure 4:**
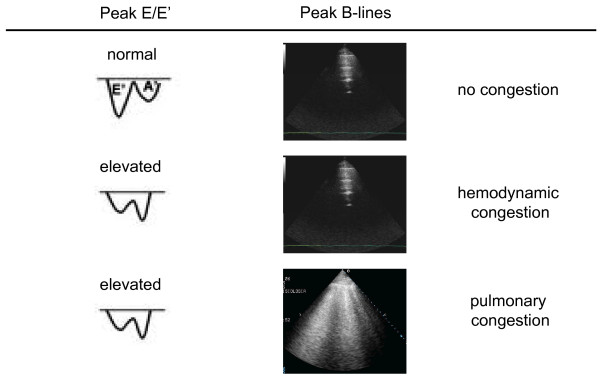
**Alveolar-capillary membrane stress echo**. The additional value of B-lines evaluation during stress echocardiography.

B-lines are very dynamic, as shown by their rapid increase after exercise, both in patients with and without left ventricular dysfunction [22]. An "alveolar-capillary stress echo" is possible by evaluation of B-lines changes during stress. They can be easily added to wall motion score index and valvular heart disease assessment during stress echocardiography, providing the additional information of the appearance of EVLW, not inferable by any other echocardiographic parameter. The presence of B-lines at peak stress can distinguish patients with stress-induced high left ventricular filling pressures but without failure of the alveolar-capillary membrane (hemodynamic congestion), from patients with stress-induced high left ventricular filling pressures and failure of the alveolar-capillary membrane, that leads to redistribution of ﬂuid within the lungs (pulmonary congestion) (figure 4).

In less than ten years, the proposal to use B-lines to evaluate pulmonary congestion in HF patients, has moved from the research setting to the clinical arena, and it is now entering recommendation papers [23,24]. Recently, it has been endorsed by a scientific statement by the Acute Heart Failure Committee of the Heart Failure Association of the European Society of Cardiology as future direction for assessing and grading congestion in acute HF [24].

In HF patients, LUS may also enable the detection of PE. Evaluation of PE is the more established application of LUS [1]. The effusion should firstly be sought in dependent zones, i.e. lateral and posterior chest. In presence of a radiopacity on chest X-ray, LUS is able to better differentiate PE from atelectasis, consolidations, masses or an elevated hemidiaphragm, and can be repeated serially at bedside. LUS has a better sensitivity and reliability than bedside chest X-ray for the diagnosis of PE [25]. Bedside chest X-ray rarely detects small effusions and can also miss effusions of up to 500 mL [26]. LUS may detect the effusion, evaluate its extension, and indicate the appropriate area for an eventual thoracentesis.

#### Treatment

The recognition, quantification and monitoring of pulmonary congestion is important for the clinician at all stages of care of the HF patient. Accurate assessment of effectiveness of medical treatment is mandatory in these patients [27]. Chest X-ray is the most used screening test for in-hospital follow-up of pulmonary congestion, although showing the above mentioned limitations. Another way to monitor congestion is through monitoring body weight. However, it has a limited reliability as a predictor of congestion status, as body weight fluctuations may not always reflect changes in intravascular volume, and weight gain may reflect normal fluctuations in time, and weight loss due to loss of muscle/fat (cardiac cachexia) may obscure increased fluid retention [28].

B-lines have been proposed as a bedside, easy-to-use, alternative diagnostic tool for clinically monitoring pulmonary congestion in HF patients [13], as they clear after adequate medical treatment. Since B-lines can be dissolved in a few minutes by an acute diuretic load, they may represent a useful bedside tool to monitor, in a real-time fashion, diuretic therapy response [29]. The dynamic behaviour of B-lines is highlighted also by their significant reduction after dialysis [30]. B-lines resolution occurs real-time as fluid is removed from the body [31], suggesting that this method could be easily employed in all situations where a dynamic evaluation of fluid changes is of importance.

The simplicity and low-techology of this examination makes it appealing also for an out-hospital office monitoring of HF patients. Pharmacological therapy could be tailored as soon as the patient, although asymptomatic, shows a significant increase in B-lines number. This could, at least in theory, prevent some new hospitalizations for worsening dyspnea, since symptoms would appear with some days of delay [32]. The possibility to assess B-lines with light, portable, hand-held devices, could also allow the cardiologist to evaluate the degree of decompensation at patients' home [15].

#### Prognosis

Persistent hemodynamic congestion, that is not adequately recognised and treated before discharge, is associated with adverse clinical outcome in HF patients [27]. On the other hand, post-discharge freedom of pulmonary congestion is associated with a better prognosis [33]. It has been demonstrated that in patients admitted to the hospital with dyspnea and/or chest pain, the presence of B-lines identifies a subgroup at higher risk of experiencing events: the higher the number of B-lines, the worse the outcome. The 16-month event-free survival showed a significantly better outcome for patients without B-lines, whereas a worse outcome was observed in patients with a severe degree of B-lines. In regard to future HF hospitalizations alone, and not as part of the combined end-point, the rate of new hospitalization for progression of HF was also higher in patients with severe B-lines and lower in patients without B-lines [34].

In patients with acute coronary syndromes, the number of B-lines, associated to some very easy echocardiographic parameters of left and right cardiac function, provide a clear prognostic stratification in a composite end-point including death, non-fatal myocardial infarction and new admission for acute decompensated HF [35].

From a practical point of view, B-lines assessment may be useful at all stages of HF management: in outpatients, to monitor increasing EVLW as a sign of impending decompensation, that is more reliable than body weight changes; for the primary diagnosis of acute HF syndromes, in patients admitted with acute dyspnea to the Emergency Room, where even an old, low-technology echographic device may allow B-lines detection; during hospitalization for risk stratification and to titrate therapies, and for prognostic stratification at discharge.

### Acute Respiratory Distress Syndrome

**Figure 5:**
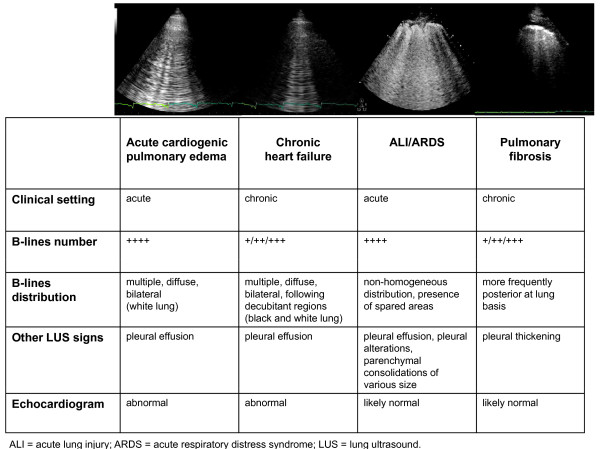
**How to distinguish different etiologies of interstitial syndrome by lung ultrasound**.

Acute respiratory distress syndrome (ARDS) is a common syndrome of diffuse lung injury with a high mortality rate [36]. Differential diagnosis between acute cardiogenic pulmonary edema and ARDS may often be difficult. In ARDS, LUS may provide a very early detection of pulmonary edema [8]. LUS showed a sensitivity of 98% and a specificity of 88% in diagnosing the presence of the interstitial syndrome as seen at CT, performing better than both auscultation and chest X-ray [37]. Being a condition of pulmonary edema, although non-cardiogenic, the sonographic pattern of multiple B-lines is present in ARDS as well as in cardiogenic pulmonary edema. However, there are some clues that may help to differentiate the two conditions, since they are often found in ARDS, but are not present in cardiogenic pulmonary edema: alterations of the pleura, due to small subpleural consolidations; "spared areas", defined as areas of normal sonographic lung appearance surrounded by areas of multiple B-lines; large consolidations of various size [38]. In patients with acute dyspnea, multiple B-lines associated to pleural alterations, represented by subpleural consolidations, are highly suggestive of non-cardiogenic pulmonary edema (Figure 5).

Although not frequently, the cardiologist may sometimes need to differentiate ARDS from cardiogenic pulmonary edema, especially in Intensive Care Unit, in patients after cardiothoracic surgery. Bedside chest X-rays are often very difficult to interpret, whereas LUS is much less affected from being performed at bedside. Moreover, LUS may be of great help in resource-limited settings, where an early diagnosis of ARDS can be life-saving, as in high altitude pulmonary edema [39,40] and after apnea diving [41].

In ARDS, LUS is useful not only in the diagnosis, but also in the follow-up: bedside LUS is able to adequately estimate lung recruitment induced by positive end-expiratory pressure (PEEP) [42], with a high significant correlation between CT and ultrasound lung reaeration scores [43].

### Pneumothorax

**Figure 6:**
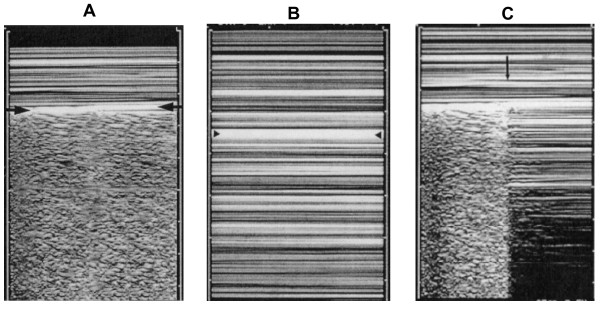
**A. Normal lung pattern on M-mode: the *seashore sign***. The motionless superficial layers generate horizontal lines (the waves). The deep artifacts follow the lung sliding, hence the sandy pattern. B. Exclusively horizontal lines are displayed, indicating complete absence of dynamics at the level of, and below, the pleural line, a pattern called the *stratosphere sign*. C. M-mode evaluation of the lung point: a sudden change from the *seashore *to the *stratosphere sign *is clearly visible (arrow). (Modified from Lichtenstein et al, 2000 [48]).

Pneumothorax (PTX) can occur after cardiothoracic surgery. Bedside chest X-ray may misdiagnose up to 30% of cases [44]. Radiographically "occult" PTX may rapidly progress to tension PTX, if its diagnosis is missed or delayed, especially in patients receiving mechanical ventilation [45]. Cardiologists may be able to diagnose PTX while performing an echocardiogram. Being a nondependent condition, in the supine patient PTX should be sought at first at the least gravitationally dependent areas, progressing more lateral. Absence of lung sliding is a basic and initial step for the diagnosis [46]. Lung sliding is the dynamic horizontal movement of the pleural line, synchronized with respiration (see additional file 1). For objectifying and documenting normal lung sliding, M-mode yields a simple pattern, the *seashore sign *(Figure 6, panel A). The presence of lung sliding allows PTX to be confidently discounted because the negative predictive value is 100% [44]. The abolition of lung sliding can be also evaluated by M-mode, which shows a characteristic pattern, the *stratosphere sign *(Figure 6, panel B), opposed to the normal *seashore sign*. However, absent lung sliding does not always mean PTX. Many other situations yield abolished lung sliding, such as high-frequency ventilation, massive atelectasis, pleural adherences, severe fibrosis, etc. Another condition needed for a LUS diagnosis of PTX is the absence of B-lines: the slightest B-line allows prompt ruling out of PTX [47]. However, the only pathognomonic LUS sign of PNX is the *lung point*, that allows PTX to be confirmed, with a specificity of 100%, and sensitivity of about 65%. Lung point is the precise area of the chest wall, where the regular reappearance of the lung sliding replaces the PTX pattern. It corresponds to the point where visceral and parietal pleura regain contact with each other. M-mode performed at the lung point, shows a clear change from one pattern to the other [48], (Figure 6, panel C).

### Acute coronary syndromes

In acute coronary syndromes, LUS should be considered as an extension of the echocardiogram, allowing in a few minutes the evaluation of pulmonary congestion, that is often difficult to be assessed with low-quality bedside chest X-rays. Evaluation of B-lines may also provide prognostic stratification [35], and may identify subjects at higher risk to develop acute pulmonary edema.

### Pulmonary fibrosis

Being a sign of thickened pulmonary interstitium, B-lines may also be present in pulmonary fibrosis [49,50]. In patients with known pulmonary fibrosis or at high risk to develop it - as in systemic sclerosis - B-lines may be an additive tool for an early detection and semiquantification of lung involvement. When aimed to evaluate pulmonary fibrosis, LUS evaluation should be focused not only on anterior and lateral chest, but also on posterior chest, because fibrotic accumulation often starts posteriorly at lung basis.

The 2 types of B-lines - cardiogenic/watery and pneumogenic/fibrotic B-lines - can pose a challenge to differential diagnosis, although some parameters may help distinguish the two entities: cardiogenic B-lines are always bilateral and are generally more diffuse on the right lung than on the left lung, with a "hot zone" of higher density along the axillary lines (in lying patients, as decubitant regions) [7], (Figure 5); moreover cardiogenic B-lines can be dissolved in a few hours by an acute diuretic load [13]. Within the clinical context of acute dyspnea, B-lines changes associated with clinical improvement, can be safely attributed to a reduction in lung water content. Other LUS signs may also help to differentiate the etiology of B-lines: in patients with congestive HF, both in acute and chronic settings, no pleural alterations are generally detectable, whereas in pulmonary fibrosis B-lines are often associated to an evident thickening of the pleura (Figure 5).

## Integrated cardio-pulmonary ultrasound evaluation

Echocardiography is an essential tool for the cardiologist, providing a huge amount of information on both acute and chronic situations. The addition of LUS to echocardiography provides an additive insight on the eventual pulmonary involvement. The cardiopulmonary system is so interconnected, that an integrated approach is mandatory. Presence of multiple, diffuse, bilateral B-lines associated to left ventricular systolic and/or diastolic dysfunction or valvular heart disease is highly indicative of cardiogenic pulmonary congestion (Figure 5). Moreover, for any given level of cardiac dysfunction, the response of the pulmonary vascular bed may be variable: LUS helps identifying those patients who, although asymptomatic, are going to decompensate and require a more aggressive treatment.

**Figure 7:**
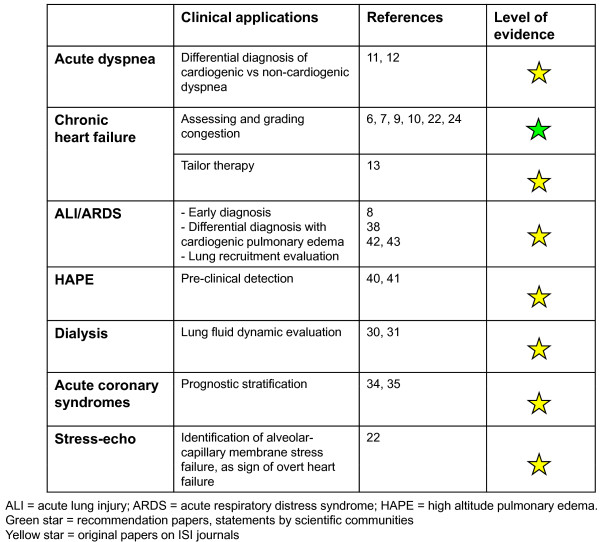
**Overview of the main clinical applications of lung ultrasound for the cardiologist**.

Presence of multiple, diffuse, bilateral B-lines, associated to a normal heart, indicates a non-cardiac cause of pulmonary edema, as acute lung injury (ALI)/ARDS, interstitial pneumonia; alternatively, especially in a chronic setting, it should pose the suspicion of pulmonary fibrosis (Figure 5). It is important to distinguish the multiple, diffuse, bilateral B-lines pattern from focal multiple B-lines, that can be present in normal lungs or may be seen around many pathologic conditions, as lobar pneumonia, pulmonary contusion, pulmonary infarction, pleural disease, neoplasia. This further underlines the importance to integrate LUS findings with patients' history, clinical presentation and other instrumental data. An overview of the main clinical applications of LUS for the cardiologist is shown in Figure 7.

LUS is one of the easiest application of echography, much easier than echocardiography. Images patterns are readily teachable, and minimal didactic and image recognition skill sessions are needed [51]. The learning curve for B-lines evaluation and grading is very short [15]. The complement of LUS to echocardiography would require only a few minutes in addition to the time needed for a resting echocardiogram.

## Limitations

LUS limitations are essentially patient dependent. Obese patients are frequently difficult to examine because of the thickness of their ribcage and soft tissues. The presence of subcutaneous emphysema or large thoracic dressings alters or precludes the propagation of ultrasound beams to the lung periphery.

The main limitation of B-lines is the lack of specificity. As already mentioned, they are a sign of interstitial syndrome, therefore they are a very sensitive but not specific sign of cardiogenic pulmonary edema. How to distinguish the different etiologies of B-lines has been discussed. However, it must be always reminded that all instrumental data should be evaluated within the clinical context and integrated with patient's history. No single test alone allows to establish the diagnosis.

## Conclusions

Providing a reliable, simple and repeatable estimation of EVLW, B-lines assessment by LUS represents a new, helpful tool for the cardiologist, to be employed at all stages of the management of HF patients, and for the differential diagnosis of dyspnea. LUS can further help in the diagnosis of other pulmonary conditions, that may be challenging in a Cardiology or Cardiac Surgery Department. Adding LUS to echocardiography may help to differentiate the main causes of acute dyspnea.

## Competing interests

The author declares that they have no competing interests.

## Supplementary Material

Additional file 1 **Sonographic pattern of the normal lung: the horizontal hyperechoic line moving synchronously with respiration is the pleura**. Click here for file

Additional file 2 **Sonographic pattern of interstitial syndrome: multiple B-lines originate from the pleural line**. Click here for file
